# Three-Dimensional Numerical Simulation of Pyrolysis of Polymethyl Methacrylate under Non-Uniform Radiative Heating

**DOI:** 10.3390/polym14245360

**Published:** 2022-12-07

**Authors:** Yujia Sun

**Affiliations:** School of Atmospheric Physics, Nanjing University of Information Science and Technology, Nanjing 210014, China; yujia.sun@nuist.edu.cn

**Keywords:** PMMA, pyrolysis, radiation, non-uniform

## Abstract

PMMA material is widely used in the building and household industries, and its pyrolysis behavior is important for fire safety. In real fire conditions, polymethyl methacrylate (PMMA) material will receive non-uniform distributed radiative heat flux from heat sources (such as fire). However, most of the existing work on this subject is limited to one dimensional geometry with uniform heat flux. This paper investigates the heat transfer and pyrolysis mechanism of PMMA material under non-uniform radiative heat flux. A three-dimensional model is developed to this end with a consideration of in-depth radiation and surface heat loss. The results show that temperature and density contours are highly non-uniform inside the solid and there is both a high-temperature core and low-density core beneath the surface. The maximum temperature occurs at a location under the top surface.

## 1. Introduction

Polymer materials are frequently used in building thermal insulation systems and indoor decorations [1]. However, their flammability poses threats to properties and life. Hence, the combustion of polymer materials is an important topic in fire science [2,3,4,5,6]. When a polymer material is exposed to a high-temperature flame, its surface will receive a large amount of heat in the form of radiation transfer and/or convective heat transfer [7,8,9,10]. The material will then experience pyrolysis and combustion. The heating and pyrolysis process is of fundamental importance for its degradation and combustion [11,12,13,14,15]. 

There have been a large amount of research focusing on the heating and pyrolysis of polymethyl methacrylate (PMMA) material. Jiang et al. [16] investigated the in-depth absorption mechanism for black PMMA, and found that in-depth radiation is the main reason for a delayed ignition time for black PMMA samples, especially under high flux conditions. Zhai et al. [17] investigated the ignition of PMMA under linearly and quadratically decreasing heat fluxes analytically and proposed an approximation model to determine the surface temperature and ignition time. Gong et al. [18] developed an analytical model to investigate the ignition mechanism of translucent polymers under exponential time-increasing heat flux. Both surface and in-depth radiation were considered. The results showed that the developed model can accurately predict surface the temperature and ignition time. Gong et al. [19] investigated the heat transfer and auto-ignition of thermally thick PMMA under linearly declining heat flux via experiments. Ignition can only occur when the decreasing rate of heat flux is lower than a critical value. Alinejad et al. [20] determined the spectral optical properties (refractive index, absorption coefficient) of black PMMA in the range of 0.25 to 25 μm and provided a set of data for the effective absorption coefficient as a function of temperature and depth. Gong et al. [21] investigated the composite auto-ignition behavior of PMMA under linearly declining radiation flux and determined the ignition criterion by the decreasing rate of heat flux. The critical mass flux was found not to be a valid criterion and a criterion considering both critical mass flux and critical temperature was proposed instead. Ding et al. [22] performed experimental and numerical simulations to study the pyrolysis of PMMA with in-depth radiation on both micro and bench scales and proved that in-depth radiation plays an important role in the numerical models for predicting temperature. 

Despite abundant research on the heat transfer and pyrolysis of PMMA materials, most existing work focusses on experimental and numerical simulation under uniform radiative heating using a 1D numerical model. To the best of the knowledge of the author, there is little work investigating pyrolysis under non-uniform radiative heat flux, which is more common in real fire conditions [23].

The purpose of present paper was to investigate the heat transfer and pyrolysis mechanism of PMMA material under non-uniform distributed radiative heat flux, which is represented by an exponentially decreasing function. The in-depth radiation absorption coefficient and convective and radiative surface heat loss were considered. Section 2 describes the numerical methods used in this paper as well as validations for the numerical methods, and Section 3 presents the most significant results of the heat transfer and pyrolysis process. Section 4 concludes this work. 

## 2. Numerical Models

### 2.1. Mathematical Model

There are various versions of governing equations for the combustion of PMMA material depending on different treatments in terms of the heating, radiation absorption and pyrolysis. Radiation heating can be applied as a surface heat flux boundary condition or a source term plugged into the energy equation. The latter method is adopted here since it is more accurate considering radiation can penetrate the solid material. The mathematical formulations for the polymers under heating and pyrolysis are:

(1) $$\begin{array}{l} \;\;\;\;\;\frac{\partial \rho }{\partial t}=-\rho S\\ \rho C\frac{\partial T}{\partial t}=k\frac{\partial^{2}T}{\partial x^{2}}+k\frac{\partial^{2}T}{\partial y^{2}}+k\frac{\partial^{2}T}{\partial z^{2}}+q_{r}\kappa e^{-\kappa z}-S△H_{s} \end{array}$$

where ρ is the density, *C* is the specific heat, *T* is the temperature, *k* is the thermal conductivity, κ is the radiation absorption coefficient, $q_{r}$ is the radiation heat flux, *S* is the evaporation/pyrolysis rate of the material, and $△H_{s}$ is the latent heat of vaporization. 

The pyrolysis rate, *S*, is:

(2) $$S=A\exp\left(-E/RT\right)$$

where *A* is the pre-exponential factor, *E* is the activation energy, and *R* is the ideal gas constant. 

The initial and boundary conditions for the energy equation are:

(3) $$T\left(x,y,z,0\right)=T_{0}$$

(4) $$-k\frac{\partial T}{\partial n}=0,\;\mathrm{side}\;\mathrm{and}\;\mathrm{bottom}\;\mathrm{walls}$$

(5) $$-k\frac{\partial T}{\partial n}=-h\left(T-T_{0}\right)-\varepsilon \sigma \left(T^{4}-T_{0}^{4}\right),\;\mathrm{top}\;\mathrm{wall}$$

The radiative heat flux takes following form:

(6) $$q_{r}=\frac{1.02\times 20000}{1+\exp\left(38.8(r-0.1)\right)}$$

where *r* is the distance to the center of the top surface. The maximum radiative heat flux is approximately 20,000 W/m^2^ at the center and then decreases towards the outside. The value 20,000 W/m^2^ was chosen here because it is very common in the literatures [24]. This equation was constructed to provide a non-uniform distributed heat flux whose value is largest at the center and decreases gradually towards outside. This distribution is typical for a material exposed to a fire [23]. 

### 2.2. Physical Model

**Figure 1:**
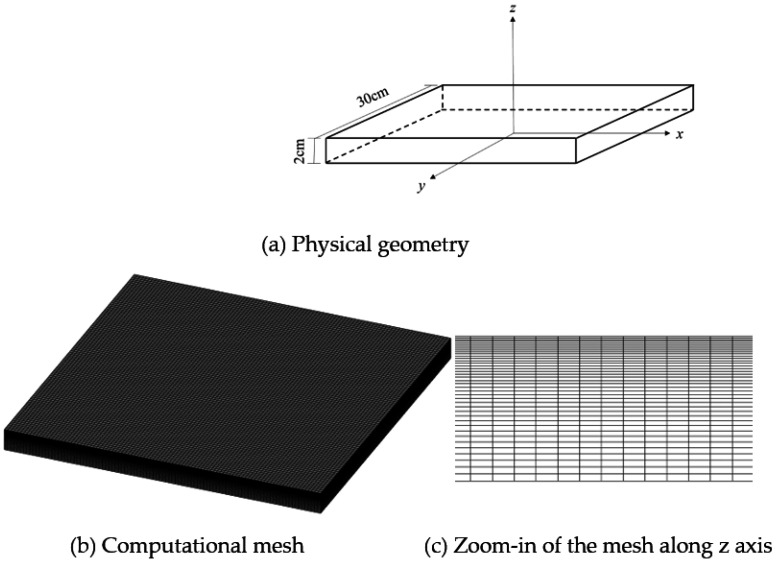
Physical geometry and computational mesh.

A cuboid geometry was considered in this work, as shown in Figure 1. The thickness of the PMMA material was 2 cm, while the length and width were 30 cm. The domain was discretized into 100-100-40 control volumes in the x, y, and z directions, respectively. As the radiative heat flux is largest near the top surface, a fine mesh was adopted in this region to capture the larger thermal gradient here, as shows in Figure 1c. 

The resulting discretized equations were solved by the finite volume method. A second-order central scheme was used for the diffusion term in the energy equation. A first order implicit scheme was used for the time discretization. The thermophysical properties needed in above equations are listed in Table 1. The values were taken from [16,24]. The temperature range considered was 300~700 K, and thermophysical properties were assumed to be constant. 

### 2.3. Validations

**Figure 2:**
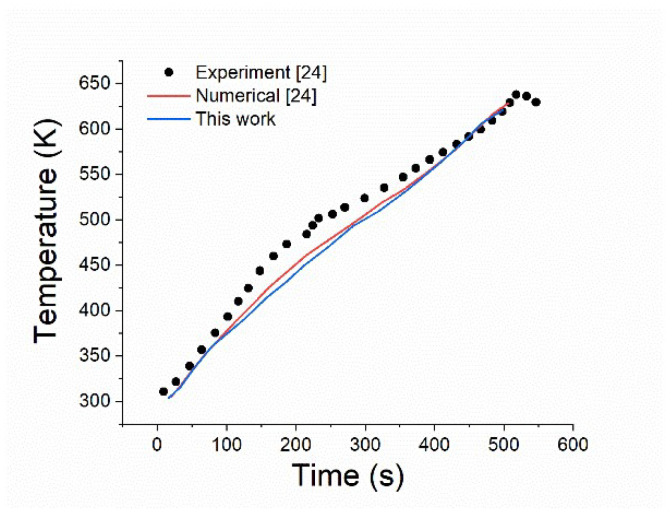
Validation of the numerical model.

To validate the accuracy of the numerical model, comparisons were made with other experiments and numerical results for a case under uniform heating. The detailed model can be found in reference [24]. Figure 2 shows the temperature history for the bottom surface of a PMMA material under uniform heating. It can be seen that the results predicted by the present method are in good agreement with the literature, proving the validity of the numerical model. 

## 3. Results and Discussions

### 3.1. Transient Temperature and Density

**Figure 3:**
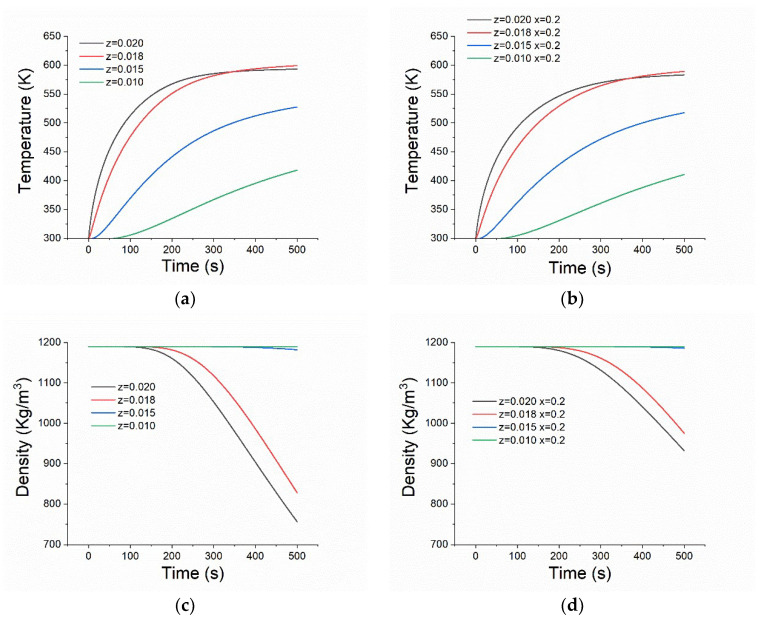
Transient temperature and density histories at different locations. (**a**) Temperature history at x = 0; (**b**) Temperature history at x = 0.2; (**c**) Density history at x = 0; (**d**) Density history at x = 0.2.

PMMA material will undergo a rise in temperature and pyrolysis due to exterior radiative heating. Figure 3 shows the transient temperature and density histories at four depth locations at the center line. “z = 0.02” represents the top surface. The surface temperature increases sharply from 0 s to 200 s at a rate about 3 K/s and then increases slowly due to the endothermic reaction. For location 2 mm (z = 0.018) below the surface, the temperature also has a large increasing rate between 0 s and 300 s but with a lag behavior. At z = 0.015 m, which is 5 mm beneath the surface, the temperature is significantly lower than those of previous locations and reaches approximately 525 K at t = 500 s. At z = 0.01 m, the temperature remains low, approximately 300 K up until 80 s and then begins to increase slowly. For the majority of the time during the heating phase, the temperature at z = 0.018 m is lower than that at z = 0.02 m due to in-depth radiation extinction. However, after 350 s, its temperature exceeds the surface temperature. This is a consequence of the combined effect of lower thermal conductivity and increased surface heat loss. Figure 3b shows the temperature histories at x = 0.2 m (0.05 m from the center). The characteristics of this location are almost the same as those of the center, and the only difference is that its temperature is slightly lower, which is due to the smaller heating flux of this region compared to the center point. 

Figure 3c,d shows the density histories at the two locations of (a) and (b). From Figure 3c, the density at the surface (z = 0.020 m) maintains its original value (1190 Kg/m^3^) for approximately 100 s, which corresponds to the heating phase of the material, and the pyrolysis is not obvious. After 100 s, the density begins to decrease slowly for a short time and then decreases almost linearly with time, reaching 750 Kg/m^3^ at t = 500 s. For location 2 mm beneath the surface (z = 0.018 m), the density has a similar trend but with a lag and a slightly lower decreasing rate. This was expected since its temperature is lower before the pyrolysis. For the locations of z = 0.015 m and z = 0.010, the density changes little within 500 s, indicating that pyrolysis has not begun. However, the density would eventually behave as that at the surface for a longer simulation time, during which the material surface regresses due to combustion. At x = 0.2 m, the density histories at four locations are almost same as those at x = 0.15 m but with a smaller decreasing rate. For example, the density at the surface is approximately 900 Kg/m^3^ at t = 500 s. 

### 3.2. Temperature Distributions

**Figure 4:**
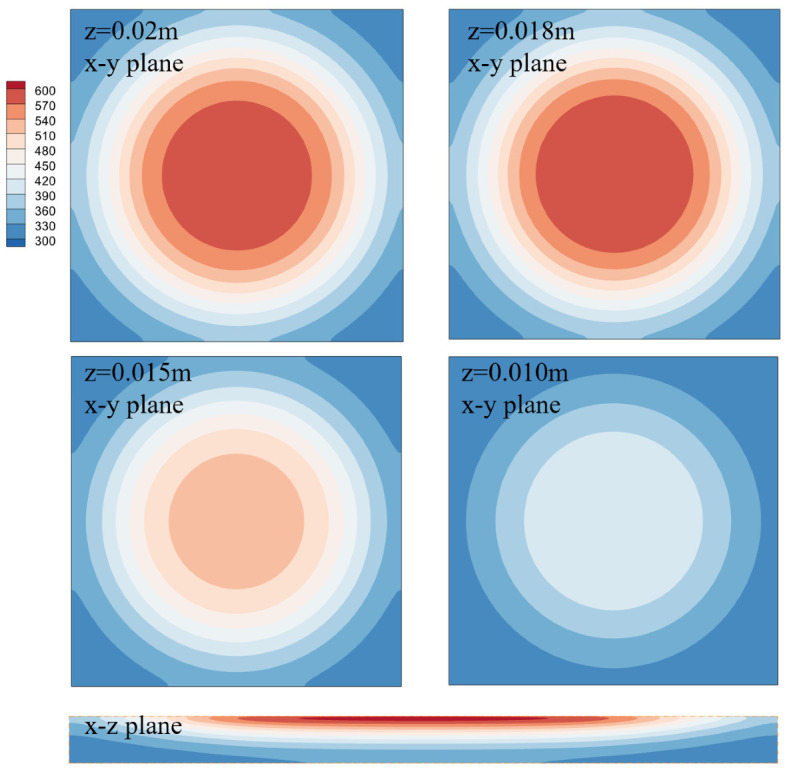
Temperature contours for different slices at t = 500 s.

Figure 4 shows the temperature distributions on the x–y plane at different depths and on the x–z plane. Four square figs correspond to the four locations in previous sections, namely, z = 0.02 m, z = 0.018 m, z = 0.015 m, and z = 0.010 m. The temperature is largest at the center region and decreases gradually towards the outside, which is consistent with the heat flux profile. The temperature distributions at z = 0.02 m and z = 0.018 m are very close to each other, while those at z = 0.015 m and z = 0.010 m are much smaller, indicating that the radiative heating mainly occurs at a thin layer near the material surface, which is caused by the large absorption coefficient of the PMMA. At an increased depth, the temperature gradient is also small compared to the surface. The rectangular contour represents the temperature on the x–z center plane. Along the depth direction, the temperature near the center penetrates further, leading to a parabolic distribution. A high temperature core region forms under the surface due to the combined effect of radiative heating, conduction, and pyrolysis. 

### 3.3. Density Distributions

**Figure 5:**
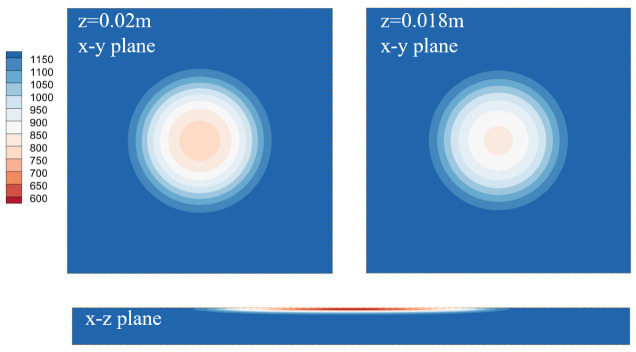
Density contours for different slices at t = 500 s.

Figure 5 shows density distributions on the x–y plane at different depths (z = 0.02 m and z = 0.018 m) and on the x–z plane. In contrast to the temperature distributions, the low-density regions have a significantly smaller sphere of influence, indicating that only the center region experiences mass loss due to pyrolysis at t = 500 s. This delay phenomenon in terms of density variation is due to the fact that pyrolysis only happens when the temperature exceeds specific values, while the temperature changes instantaneously when there is a thermal gradient under the assumption of the infinite velocity of the thermal wave. From the rectangular contour, the density distribution on the x–z plane has a very thin layer of low values, whose influence is much smaller than the temperature. For this low-density region, the density becomes smallest near the surface center and then decreases gradually towards the outside and inside. 

**Figure 6:**
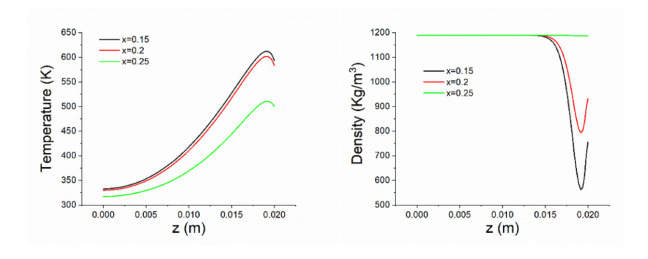
Temperature and density profiles along the depth at different locations.

To clearly show the temperature and density distribution along the depth direction, Figure 6 show their profiles along the z axis for three locations, x = 0.15 m (surface center), x = 0.2 m, and x = 0.25 m at t = 500 s. It is obvious that the surface temperature is not the largest at this time. Along the depth direction, the temperature first increases and then decreases gradually to lower values. The highest temperature occurs near z = 0.018 m, and the maximum value decreases towards the outside. Accordingly, the lowest density occurs along the depth direction. For x = 0.15 m, the density at the surface is approximately 750 Kg/m^3^, while the lowest value at this location is less than 600 Kg/m^3^ near z = 0.018 m-z = 0.019 m. The differences between the density profiles are larger than those for the temperature. 

## 4. Conclusions

A three-dimensional numerical simulation was performed to investigate the heating and pyrolysis behavior of a PMMA material under non-uniform radiative heating. The in-depth radiation and surface heat loss were considered. The numerical model was validated by a case under uniform radiative heating, and the result predicted by the present work is in good agreement with those of experimental and other numerical simulations, proving the accuracy of the numerical model. For the non-uniform heating case, the radiative heat flux penetrating the PMMA was represented by an exponentially decreasing function. The results show that the temperature at the surface will initially increase sharply and then slowly and that there is a thin layer with a high temperature. The density near the surface decreases with time, with a lag in relation to the temperature rise. The temperature and density contours are highly non-uniform inside the PMMA. Due to the combined effect of pyrolysis, surface heat loss, and conduction, the maximum temperature occurs at the location beneath the surface with the lowest density. 

## Figures and Tables

**Table 1:** Thermophysical properties for the PMMA materials.

Parameter	Value	Units
*k*	0.188	W/m K
*C*	1465	J/kg K
*ρ*	1190	Kg/m^3^
*κ*	960.5	m^−1^
*ε*	0.96	
*h*	10	W/m^2^ K
A	8.6 × 10^12^	s^−1^
E	1.88 × 10^5^	J/mol
$△H_{s}$	846	J/g

## Data Availability

The data presented in this study are available on request from the corresponding author.
